# Monitoring Occupational Sitting, Standing, and Stepping in Office Employees With the W@W-App and the MetaWearC Sensor: Validation Study

**DOI:** 10.2196/15338

**Published:** 2020-08-04

**Authors:** Judit Bort-Roig, Emilia Chirveches-Pérez, Francesc Garcia-Cuyàs, Kieran P Dowd, Anna Puig-Ribera

**Affiliations:** 1 Sport and Physical Activity Research Group Centre for Health and Social Care Research University of Vic-Central University of Catalonia Vic Spain; 2 Research Group on Methodology, Methods, Models and Outcomes of Health and Social Sciences Centre for Health and Social Care Research University of Vic-Central University of Catalonia Vic Spain; 3 Digital Care Research Group Centre for Health and Social Care Research University of Vic-Central University of Catalonia Vic Spain; 4 Department of Sport and Health Sciences Athlone Institute of Technology Athlone Ireland

**Keywords:** validity, self-monitoring, sedentary behavior, physical activity, smartphone, mobile phone, device-based measure

## Abstract

**Background:**

Replacing occupational sitting time with active tasks has several proposed health benefits for office employees. Mobile phones and motion sensors can provide objective information in real time on occupational sitting behavior. However, the validity and feasibility of using mobile health (mHealth) devices to quantify and modify occupational sedentary time is unclear.

**Objective:**

The aim of this study is to validate the new Walk@Work-Application (W@W-App)—including an external motion sensor (MetaWearC) attached to the thigh—for measuring occupational sitting, standing, and stepping in free-living conditions against the activPAL3M, the current gold-standard, device-based measure for postural behaviors.

**Methods:**

In total, 20 office workers (16 [80%] females; mean age 39.5, SD 8.1 years) downloaded the W@W-App to their mobile phones, wore a MetaWearC sensor attached to their thigh using a tailored band, and wore the activPAL3M for 3-8 consecutive working hours. Differences between both measures were examined using paired-samples *t* tests and Wilcoxon signed-rank tests. Agreement between measures was examined using concordance correlation coefficients (CCCs), 95% CIs, Bland-Altman plots (mean bias, 95% limits of agreement [LoA]), and equivalence testing techniques.

**Results:**

The median recording time for the W@W-App+MetaWearC and the activPAL3M was 237.5 (SD 132.8) minutes and 240.0 (SD 127.5) minutes, respectively (*P*<.001). No significant differences between sitting (*P*=.53), standing (*P*=.12), and stepping times (*P*=.61) were identified. The CCC identified substantial agreement between both measures for sitting (CCC=0.98, 95% CI 0.96-0.99), moderate agreement for standing (CCC=0.93, 95% CI 0.81-0.97), and poor agreement for stepping (CCC=0.74, 95% CI 0.47-0.88). Bland-Altman plots indicated that sitting time (mean bias –1.66 minutes, 95% LoA –30.37 to 20.05) and standing time (mean bias –4.85 minutes, 95% LoA –31.31 to 21.62) were underreported. For stepping time, a positive mean bias of 1.15 minutes (95% LoA –15.11 to 17.41) was identified. Equivalence testing demonstrated that the estimates obtained from the W@W-App+MetaWearC and the activPAL3M were considered equivalent for all variables excluding stepping time.

**Conclusions:**

The W@W-App+MetaWearC is a low-cost tool with acceptable levels of accuracy that can objectively quantify occupational sitting, standing, stationary, and upright times in real time. Due to the availability of real-time feedback for users, this tool can positively influence occupational sitting behaviors in future interventions.

**Trial Registration:**

ClinicalTrials.gov NCT04092738; https://clinicaltrials.gov/ct2/show/NCT04092738

## Introduction

Replacing sedentary time (ie, sitting, lying, or reclining postures that involve an energy expenditure of ≤1.5 metabolic equivalent units during waking hours) [1] with physical activity (PA) or movement of any kind has proposed health benefits for adults [2]. Positive associations have been reported with cardiometabolic biomarkers, mortality risk reduction, and body composition [3]. Many adults accumulate large amounts of daily sitting time at work, with white-collar workers being the most likely to engage in extensive occupational daily sitting [4]. Given that leveraging the time-inverse relationship between sedentary behaviors (SB) and PA could achieve important public health benefits [5], interventional efforts should target this high-risk subgroup [6] in the setting where daily sitting mostly occurs [5].

Self-monitoring is a key element to increase individuals’ awareness of and empowerment toward behavior change [7]. For PA and SB, self-reported questionnaires have traditionally been the most commonly employed tool in largescale population studies due to their low cost, simplicity, and feasibility [8-11]. However, technological advances over the last 2 years have enabled the use of device-based measures, such as accelerometers, for self-monitoring PA and SB [8].

Evidence has identified mobile phones as a potential alternative to accurately self-monitor PA and SB via inbuilt inertial sensors [12-15]. However, battery life and mobile phone location have been major issues that have compromised usability and long-term monitoring. While external devices, such as wearables, may have overcome such weaknesses [16], the most popular devices are commercial motion sensors that use acceleration data to recognize activity behaviors (ie, distance, time, and intensity). Unfortunately, such measures struggle to distinguish postures (ie, sitting and standing), primarily due to wear position (ie, where the device is placed) and the use of proprietary algorithms that do not accurately quantify such behaviors [7].

Commercially available devices that examine SB through postural positioning rather than lack of movement (ie, acceleration) are scarcer [17]. However, devices that quantify time spent sitting, standing, and light intensity PA are critical when self-monitoring occupational behaviors, as moderate-to-vigorous physical activity is less prevalent during working hours or transport time to and from work [18].

Mobile phones alone struggle with postural identification due to the nonattachment of phones to the body and the ubiquitous nature of phone use [12]. However, the use of mobile phones with external monitoring devices may have the potential to become an accurate, cost-effective self-monitoring tool [12]. The range of novel and engaging mobile phone–based intervention strategies, as well as the user’s perceptions on their usefulness and viability, highlights the potential of such technology on PA promotion [12].

In this context, the Walk@Work-Application (W@W-App) was developed to self-monitor occupational PA and SB with a high level of validity. The W@W-App communicates with a MetaWearC external sensor [19], attached via a band to the thigh, to quantify occupational sitting, standing, and stepping while offering real-time feedback on these behaviors, which is an essential component for changing behaviors at the time and place where they occur. This study examined the validity of the W@W-App+ MetaWearC tool to quantify time spent in occupational sitting, standing, and stepping against the current gold-standard, device-based measure for postural behaviors.

## Methods

### Measurement Tools

**Figure 1:**
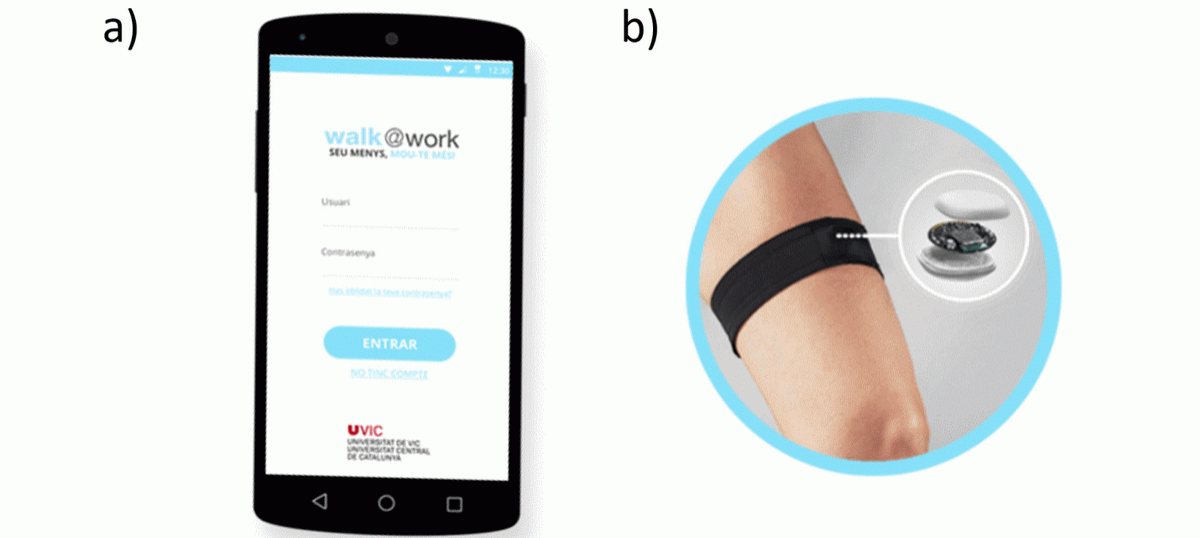
(A) Initial W@W-App interface and (B) the MetaWearC sensor with the waterproof case and the thigh band.

The new W@W-App was developed from a previous version [20], adding a commercially available sensor (MetaWearC; MbientLab Inc) to gather postural and movement information. The MetaWearC is a small sensor (24 mm × 6 mm; 5.6 g) covered with a waterproof round case. The sensor is a triaxial accelerometer with an amplitude range of ±16 g and a sampling rate of 6.25 Hz. Key features of the MetaWearC sensor are shown on the MbientLab web page [19]. Raw sensor data are synchronized with the W@W-App software via a low-energy Bluetooth system with a long battery life (>30 days) and a range of up to 10-15 meters. The data are directly processed and displayed in real time by the app on the phone and securely stored on the backend server. Figure 1 depicts the W@W-App (login page) and the MetaWearC sensor.

**Figure 2:**
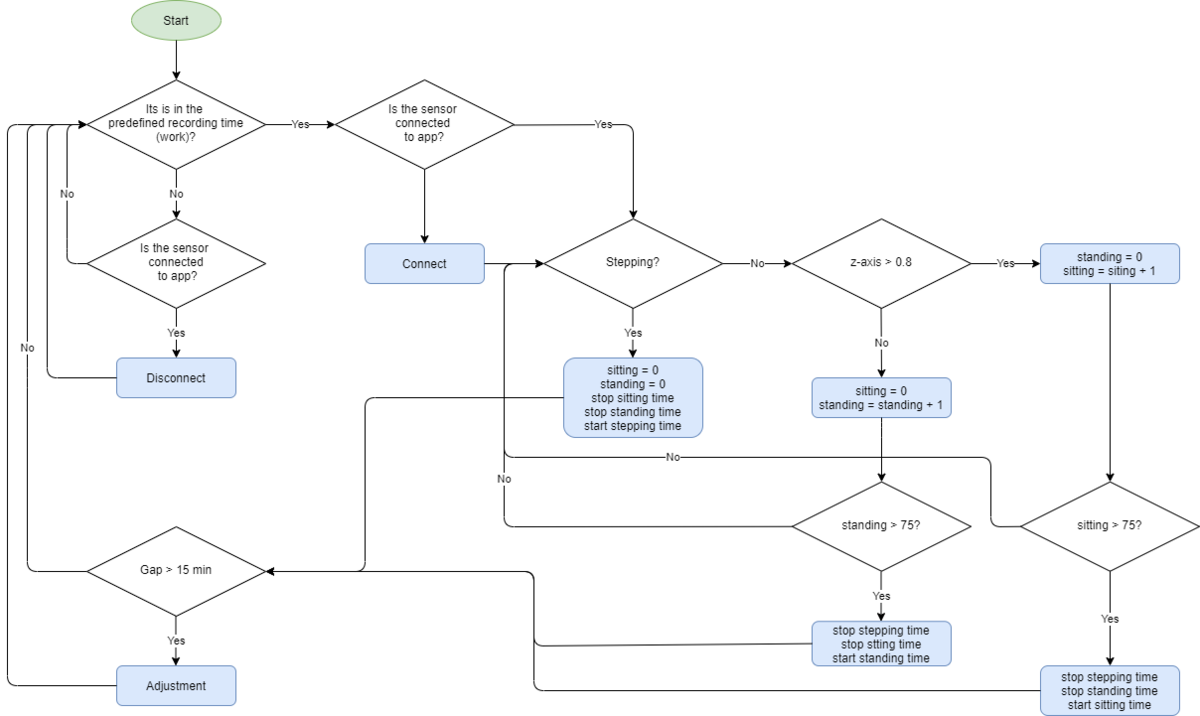
Flow diagram illustrating the algorithm for decision-rules used by the W@WApp + MetaWearC sensor to determine sitting, standing and stepping time.

The algorithm for the W@W-App+MetaWearC (Figure 2) was designed to analyze accelerometer output from the MetaWearC sensor. The MetaWearC sensor is worn within a small bag inside an elastic and adjustable band (Figure 1) that is attached to the participant’s right thigh. The algorithm is based on two primary requirements: (i) data can only be recorded during the defined recording period (ie, working hours) and (ii) data can only be collected when both the device and the software are connected via Bluetooth. When these criteria have been met and the sensor detects an acceleration, the step counter begins and the sitting and standing counters are reset to 0. Stepping time is initiated when the sensor identifies a balance between false positives (ie, counting a step when the step has not happened) and false negatives (ie, not counting a step when the step has occurred). There are three sensitivity modes for the step detector: normal, sensitive, and robust. These modes balance sensitivity (false negatives) and robustness (false positives). Normal mode is used in most applications as it provides a balance between false positives and false negatives. An example of a false positive would be the detection of a step while an individual is in a sitting position, possibly as a result of stretching one’s leg.

The recognition of postures (sitting and standing) is based on the angle of the z-axis, where 0 indicates a completely vertical posture (standing) and 1 indicates a completely horizontal posture (sitting). When the sensor detects a value higher than 0.8 in the z-axis, the sitting counter is initiated while the standing counter remains to 0. When the sensor detects a value equal to or lower than 0.8 in the z-axis, the standing counter begins while the sitting counter returns to 0. If either the sitting or standing counters reach 75 readings (approximately every 2 seconds), this indicates that the sensor has not detected stepping during those 75 readings, and therefore the step counter stops and assumes that the user is either sitting or standing depending on which of these counters reaches 75.

Finally, if there is a difference greater than 15 minutes between the time counters for the W@W-App-MetaWearC (stepping, sitting, and standing) and the elapsed time, a weighted adjustment is completed. Normally, this difference is due to temporal disconnections of the sensor if it is kept more than 20 meters away from the mobile phone. For example, if the W@W-App-MetaWearC has counted for 100 minutes (75 minutes stepping, 10 minutes standing, and 15 minutes sitting), but the real time elapsed is 115 minutes, the weighted adjustment will correct the W@W-App-MetaWearC to 86 minutes stepping, 12 minutes standing, and 17 minutes sitting.

The activPAL3M (PAL Technologies Ltd) is referred to as the gold-standard, device-based measure for postural recognition in free-living conditions [21]. The activPAL3M was employed as the criterion measure for sitting, standing, and stepping times. The activPAL3M (25 mm × 45 mm × 5mm; 9g) was placed in a waterproof nitrile sleeve and attached on the midline of the anterior aspect of the participant’s thigh using a transparent film (10 cm × 10cm of hypoallergenic Tegaderm Foam Adhesive Dressing).

### Participants and Procedures

Office workers from the University of Vic-Central University of Catalonia (UVic-UCC) who owned a mobile phone (Android version 6.0.0/iOS version 10.0.0 or higher) were invited to participate in the study. A convenience sample was recruited (N=23). All volunteers provided written informed consent prior to participation. This study was conducted within a Spanish national project (W@WApp-Diab; PI17/01788) led by the UVic-UCC. Ethical approval was obtained by the research ethics committee of the Research Institute of Primary Care Jordi Gol (IDIAP).

Participants installed and configured the W@W-App, following guidance provided by the researchers: (i) registration on the Walk at Work web platform [22], (ii) user verification through email, (iii) W@W-App installation and initialization, (iv) recording day and time period configuration (ie, between 3 and 8 working hours), and (v) recognition of the MetaWearC sensor via Bluetooth. In adherence to the European Union General Data Protection Regulation, participants could read the private policy of the W@W-App, which is written using clear and straightforward language, on the Walk at Work website [22]. In addition, participants provided affirmative consent prior to using the W@W-App when they voluntarily registered on the web platform.

Researchers initialized the activPAL3M and the W@W-App+MetaWearC and placed both devices on the midpoint (ie, one over the other) of the anterior aspect of the thigh of the same leg to avoid measurement bias due to asymmetric leg positions and movements. To ensure that the timestamp of the W@W-App+MetaWearC and the activPAL3M aligned for data analysis, they were initialized from the same PC.

Participants wore the W@W-App+MetaWearC and the activPAL3M sensor in occupational free-living conditions for 3-8 hours. They were required to keep their mobile phone within a 5-meter radius throughout the measurement period (ie, participants were asked to keep their mobile phones with them at all times).

### Variables and Statistical Analysis

The variables recorded and quantified by the W@W-App+MetaWearC were time spent in sitting and standing postures and time spent stepping. These variables were extracted from the W@W-App software. For the activPAL3M, files were processed via the activPAL Professional Software (version 7.2.32) upon completion of data collection. Data were then exported to a Microsoft Excel (Microsoft Excel 2016; Microsoft Corporation) file format, providing data on sitting, standing, and stepping in 15-second epochs. This enabled the quantification of the number of minutes spent sitting, standing, and stepping. In addition, the time spent sitting and standing were added together to quantify stationary time, while the amount of time spent standing and stepping were added together to compute upright time. Total recording time (ie, minutes) from both devices was calculated by summing the amount of time spent sitting, standing, and stepping.

Descriptive characteristics (mean [SD] and median [IQR]) were used to describe the data. Differences between the W@W-App+MetaWearC and the activPAL3M were examined using paired-samples *t* tests and Wilcoxon signed-rank tests. Pearson correlation coefficients were used to determine the strength and direction of association between variables quantified by the two measures when the data were normally distributed. Spearman rank-order correlation coefficients were employed when data were not normally distributed. The concordance correlation coefficient (CCC), using Lin’s approach [23], was used to examine the level of agreement between the W@W-App+MetaWearC variables and the activPAL3M determined variables. The CCC values were interpreted using the categorization recommended by McBride [24]. Bland-Altman plots with mean bias and limits of agreement (LoA) were constructed to examine the agreement between the W@W-App+MetaWearC variables and the activPAL3M determined variables using similar approaches reported previously [25]. Equivalence was determined using two one-sided paired *t* tests (90% CI) for the mean difference between the W@W-App+MetaWearC variables and the activPAL3M determined variables [26]. Equivalence was supported if the CI for the mean difference was within 15% of the activPAL3M-determined time spent sitting, standing, and stepping. The equivalence region was arbitrarily defined, as limited biologically and analytically relevant criteria can be defined for the equivalence regions for sitting, standing, and stepping. Less conservative equivalence regions were also tested in case the equivalence was not supported for the 10% level. Additional tests to determine the region of equivalence were completed using increments of 5%. This approach was selected to provide a clear estimation of the accuracy of the W@W-App+MetaWearC [27]. Measures were expected to differ by no more than 30 minutes for sitting, 11 minutes for standing time, and 4 minutes for stepping. All statistical analysis was conducted using IBM SPSS Statistics 25 (IBM Corporation) and Microsoft Excel (Microsoft Corporation).

## Results

In total, 23 office workers participated in the study, whereby activity behavior information was recorded by both measures during workplace free-living conditions between October and November 2018. After excluding 3 participants because of technical problems with the mobile phone, data from 20 participants were included in the analyses (age: mean 39.5 years, SD 8.1, range 27-60; 16 [80%] women). A total of 115 hours of data was recorded, with an average of 5 hours per participant. Of all participants, 13 used an Android smartphone (Samsung, n=5; BQ Aquaris, n=4; Xiaomi, n=2; Xperia, n=1; and Huawei, n=1) with an operational system version ranging from 6.0.1 to 8.0.0. The other 7 participants employed an iPhone 6 or iPhone 7 with an operational system version higher than 10.3.3.

Descriptive characteristics for variables of interest from the W@W-App+MetaWearC and the activPAL3M, as well as the statistical differences between the two measures for each variable, are described in Table 1. The median recording time for the W@W-App+MetaWearC was 237.50 (SD 132.75) minutes, while the activPAL3M median recording time was 240.00 (SD 127.50) minutes. No significant differences between the W@W-App+MetaWearC and the activPAL3M were observed for sitting time (*P*=.53), standing time (*P*=.12), and stepping time (*P*=.54).

**Table 1:** Descriptive characteristics and statistical significance (*P* value) of the differences between the W@W-App+MetaWearC and the activPAL3M for minutes spent in different activity behaviors (N=20).

Variable	W@W-App	activPAL3M	*P* value
Recording time (min), median (IQR)	237.5 (132.8)^a^	240.0 (127.5)^a^	<.001
Sitting time (min), median (IQR)	191.0 (132.0)^a^	180.5 (124.3)^a^	.53
Standing time (min), mean (SD)	70.3 (38.1)	75.4 (36.1)	.12
Stepping time (min), median (IQR) or mean (SD)	22.0 (24.0)^a^	24.0 (10.5)	.61
Stationary time (min), median (IQR)	223.5 (147.3)^a^	227.0 (138.0)^a^	.002
Upright time (min), mean (SD)	47.7 (23.6)	49.7 (21.8)	.25

^a^Data presented as median (IQR) due to nonnormality.

The W@W-App+MetaWearC showed strong to very strong correlations with activPAL3M-determined activity variables. CCCs identified substantial agreement between the two measures for sitting (CCC=0.98, 95% CI 0.96-0.99), moderate agreement for standing (CCC=0.93, 95% CI 0.81-0.97), and poor agreement for stepping (CCC=0.74, 95% CI 0.47-0.88). The correlation coefficients, CCC values, and associated 95% CI are shown in Table 2.

**Table 2:** Correlation coefficients (*r*), concordance correlation coefficients (CCC), and 95% CIs between the W@W-App+MetaWearC and the activPAL3M for minutes spent in different activity behaviors (N=20). All *P*<.001.

Variable	*r* (95% CI)	CCC (95% CI)
Recording time (min)	0.89 (0.73-0.95)	0.99 (0.99-0.99)
Sitting time (min)	0.97 (0.92-0.99)	0.98 (0.96-0.99)
Standing time (min)	0.93 (0.83-0.97)	0.92 (0.82-0.97)
Stepping time (min)	0.74 (0.44-0.89)	0.74 (0.47-0.88)
Stationary time (min)	0.96 (0.90-0.98)	0.99 (0.99-1.00)
Upright time (min)	0.95 (0.88-0.98)	0.95 (0.87-0.98)

**Figure 3:**
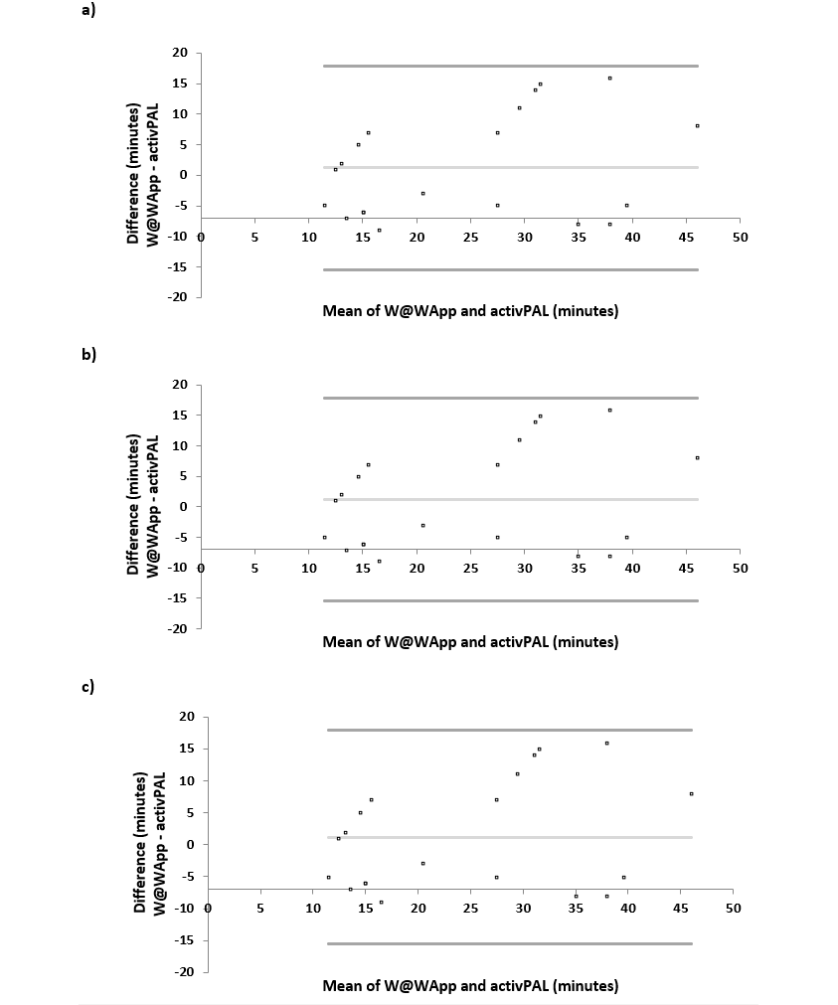
Bland-Altman plots of absolute agreement for (A) sitting, (B) standing, and (C) stepping, derived from the W@W-App+MetaWearC with the equivalent outcome derived from the activPAL3M.

**Figure 4:**
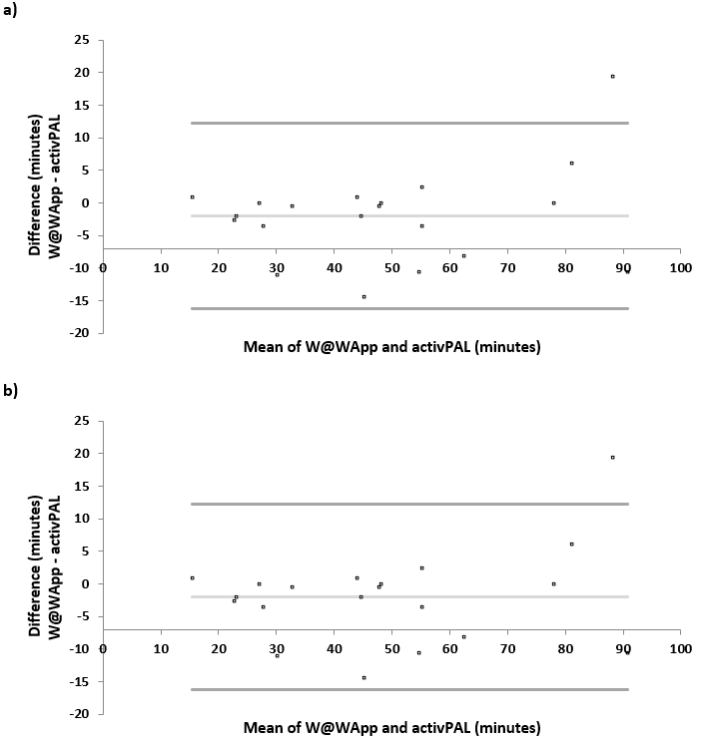
Bland-Altman plots of absolute agreement for (A) stationary time (sitting+standing) and (B) upright time (standing+stepping), derived from the W@W-App+MetaWearC with the equivalent outcome derived from the activPAL3M.

The mean bias and LoA from the Bland-Altman analysis are provided in Table 3. The Bland-Altman plots, which compare the mean sitting, standing, stepping, stationary, and upright times measured by the W@W-App+MetaWearC and the activPAL3M are presented in Figures 3 and 4. The Bland-Altman plots present a graphical description of the means for sitting, standing, stepping, stationary, and upright times as measured by the W@W-App+MetaWearC and the activPAL3M against the difference of the time spent in each of these behaviors between both measures. For sitting, a smaller mean bias was observed (–1.66 minutes) with a relatively wide LoA (–30.37 to 27.05). The equivalence procedure indicated that the 90% CI for the mean difference was 0.2 and 20.8 and was within the 15% equivalence region (–30.0 to +30.0 minutes). The estimates obtained from the two measures were considered equivalent for sitting time. The largest observed mean bias for a specific behavior was observed for standing time (–4.85 minutes; LoA –31.31 to 21.62). The 90% CI for the mean difference was –10.5 and 0.3 and was within the 15% equivalence region (–11.0 to +11.0 minutes). The estimates obtained from the two measures were considered equivalent for standing time. For stepping time, a small mean bias was observed (1.15 minutes; LoA –15.11 to 17.41). However, the equivalence procedure indicated that the 90% CI for the mean difference was –4.5 and 2.1, which was not significantly within the 15% equivalence region (–4.0 to +4.0 minutes). The estimates obtained from the two measures were not considered equivalent for stepping time.

**Table 3:** Mean bias and limits of agreement (LoA) for sitting, standing, and stepping times.

Variable	Mean bias	Lower LoA	Upper LoA
Recording time (min)	–5.37	–13.56	2.81
Sitting time (min)	–1.66	–30.37	27.05
Standing time (min)	–4.85	–31.31	21.62
Stepping time (min)	1.15	–15.11	17.41
Stationary (min)	–6.52	–20.81	7.78
Upright time (min)	–1.85	–15.76	12.06

When combining variables, stationary time significantly differed between the two measures (*P*=.002), while no differences were observed for upright time (*P*=.25). However, stationary and upright times were strongly correlated with the criterion measure (activPAL3M) (*P*<.001). Time spent on stationary activities was underestimated with a mean bias of –6.52 minutes, with a relatively small LoA (–20.81 to 7.78 minutes). The 90% CI for the mean difference was 1.3 and 12.4 and was within the 15% equivalence region (–41 to +41 minutes). The estimates obtained from the two measures were considered equivalent for stationary time. A mean bias of –1.85 minutes was identified for time spent upright, with a relatively small LoA (–15.76 to 12.06 minutes) compared to the noncombined postural/activity variables. The equivalence procedure indicated that the 90% CI for the mean difference was –0.9 and 4.8 and was within the 15% equivalence region (–7.0 to +7.0 minutes). The estimates obtained from the two measures were considered equivalent for upright time.

## Discussion

### Principal Findings

This study examined the validity of the W@W-App+MetaWearC to measure occupational sitting, standing, and stepping times in a free-living workplace environment. Our findings indicated that the W@W-App+MetaWearC is a valid tool for self-monitoring occupational sitting, standing, stationary, and upright times, demonstrating moderate to very strong validity when compared to the criterion measure (activPAL3M). However, the analysis demonstrated that the findings for stepping from the W@W-App+MetaWearC are not equivalent to those from the activPAL3M.

Although a small mean bias of 1.15 minutes for stepping was observed between the W@W-App+MetaWearC and the activPAL3M, poor agreement, wide CIs and nonequivalence would suggest that the W@W-App+MetaWearC should not be recommended for use in detecting stepping time. However, it is quite plausible that these observations can be attributed not only to variance from the W@W-App+MetaWearC tool but also variance in the activPAL3M device. The activPAL3M is primarily used to examine postural position, namely sitting and standing, and has demonstrated high levels of accuracy in the detection of these behaviors in lab-based and free-living conditions [28], justifying its use as a device-based comparison for the measurement of sitting and standing times. However, lower levels of validity for the activPAL3M have been highlighted for stepping time and step count, particularly during activities of daily living. Therefore, future research should aim to utilize more accurate methods of movement when validating the W@W-App+MetaWearC tool. It should be acknowledged that W@W-App+MetaWearC performs relatively well in the detection of steps in free-living conditions when compared with findings from other commercially available activity monitors [29,30].

For stationary time (ie, sitting and standing), the W@W-App+MetaWearC demonstrated high levels of accuracy when compared with previous validation studies employing a range of activity monitors [31]. This is likely due to the ability of the W@W-App+MetaWearC to detect sitting and standing postures based on thigh acceleration. Recent studies have developed and validated self-monitoring devices that also provide real-time feedback on an integrated display, including the SitFit [32] or through a mobile phone app via Bluetooth synchronization such as the VitaBit [33] and Chair&App [34]. Similar to the findings presented here, the SitFit and Chair&App devices reported that sitting time was highly accurate when compared to the activPAL3M in free-living conditions. However, the W@W-App+MetaWearC reported a lower mean bias (W@W-App+MetaWearC) in comparison to other studies (SitFit). In contrast, the VitaBit device did not accurately distinguish between sitting and standing in free-living conditions but was accurate in the detection of movement. These findings are unsurprising, since the device used as the comparison measure (ActiGraph) struggles to accurately distinguish sitting and standing behaviors [35]. Both the SitFit and the VitaBit were designed to be worn in the pocket of a user’s trousers, which may be a usability barrier when wearing clothes without pockets. Chair&App, as well as the W@W-App, focused on office-based jobs, but Chair&App used a regular office chair equipped with pressure sensors instead of a thigh-attached device. That may remove compliance issues related to recording time but standing, stepping, and sitting away from one’s personal desk cannot be captured. The W@W-App has demonstrated high levels of validity for sitting, standing, stationary, and upright times, while the wearer’s location and attachment site may increase compliance with wearing a self-monitoring tool in the workplace.

The W@W-App+MetaWearC is a novel tool that simulates the activPAL3M activity monitor in accurately recognizing postural position at the workplace. The output from the W@W-App+MetaWearC tool for sitting, standing, stationary, and upright times were identified as equivalent to the current gold-standard, device-based postural measure, the activPAL3M. This suggests that this self-monitoring tool, which provides real-time feedback to users, is worthwhile for use in interventions that aim to reduce sitting behavior in the workplace. Self-monitoring is vital for increasing individuals’ awareness and empowerment toward behavior change [7]. This may result in a more accurate, affordable, and accessible device than those currently available, enabling the more cost-effective inclusion of SB self-monitoring as a function of SB interventions in the future.

### Strengths and Limitations

The strengths of this study include (i) the examination of the complete range of occupational sedentary and activity behavior types (sitting/lying, standing, and stepping), (ii) the examination of the validity of these measures in occupational free-living conditions, and (iii) the use of a gold-standard, objective measurement device to determine the validity of the W@W-App+MetaWearC.

The present study is not without limitations. Although the activPAL3M has been described as the gold standard for measuring sitting time [21] and is an acceptable field-based measure for activity behaviors in youth and adult populations [36,37], it is not the gold standard for comparison of stepping time. This should then be considered when interpreting the Bland-Altman plots, as these are designed to support the comparison of a new measure to a previous gold standard. The relatively small sample size with a large percentage of females (16/20) and the homogeneity of the workplace setting (ie, all sampled from a university context) might differ from the general office population. Furthermore, the data gathered included an average of 5 hours per subject, providing a limited timeframe. Additionally, the wide range of operating systems and hardware available added complexity to app development and subsequent validation.

### Conclusions

The W@W-App+MetaWearC self-monitoring system demonstrates high levels of accuracy in determining postural position. This tool is a low-cost alternative tool for the examination of occupational sitting and standing times. It has demonstrated high levels of validity in detecting postural position and provides real-time feedback to users. Future research should examine the interventional effect of utilizing this system as a self-monitoring tool for modifying activity behaviors in office-based workers.

## Abbreviations

CCC: concordance correlation coefficient
LoA: limits of agreement
mHealth: mobile health
PA: physical activity
UVic-UCC: University of Vic-Central University of Catalonia
W@W-App: Walk@Work-Application
